# Identification of candidate genes involved in coronary artery calcification by transcriptome sequencing of cell lines

**DOI:** 10.1186/1471-2164-15-198

**Published:** 2014-03-14

**Authors:** Shurjo K Sen, Jennifer J Barb, Praveen F Cherukuri, David S Accame, Abdel G Elkahloun, Larry N Singh, Shih-Queen Lee-Lin, NISC Comparative Sequencing Program, Frank D Kolodgie, Qi Cheng, XiaoQing Zhao, Marcus Y Chen, Andrew E Arai, Eric D Green, James C Mullikin, Peter J Munson, Leslie G Biesecker

**Affiliations:** 1 National Human Genome Research Institute, National Institutes of Health, Bethesda, MD 20892, USA; 2 Mathematical and Statistical Computing Laboratory, Center for Information Technology, National Institutes of Health, Bethesda, MD 20892, USA; 3 CVPath Institute, Inc, Gaithersburg, MD 20878, USA; 4 National Heart Lung and Blood Institute, National Institutes of Health, Bethesda, MD 20892, USA

**Keywords:** Coronary artery calcification, RNA-Seq, Lymphoblastoid cell lines, Transcriptome profiling

## Abstract

**Background:**

Massively-parallel cDNA sequencing (RNA-Seq) is a new technique that holds great promise for cardiovascular genomics. Here, we used RNA-Seq to study the transcriptomes of matched coronary artery disease cases and controls in the ClinSeq® study, using cell lines as tissue surrogates.

**Results:**

Lymphoblastoid cell lines (LCLs) from 16 cases and controls representing phenotypic extremes for coronary calcification were cultured and analyzed using RNA-Seq. All cell lines were then independently re-cultured and along with another set of 16 independent cases and controls, were profiled with Affymetrix microarrays to perform a technical validation of the RNA-Seq results. Statistically significant changes (p < 0.05) were detected in 186 transcripts, many of which are expressed at extremely low levels (5–10 copies/cell), which we confirmed through a separate spike-in control RNA-Seq experiment. Next, by fitting a linear model to exon-level RNA-Seq read counts, we detected signals of alternative splicing in 18 transcripts. Finally, we used the RNA-Seq data to identify differential expression (p < 0.0001) in eight previously unannotated regions that may represent novel transcripts. Overall, differentially expressed genes showed strong enrichment (p = 0.0002) for prior association with cardiovascular disease. At the network level, we found evidence for perturbation in pathways involving both cardiovascular system development and function as well as lipid metabolism.

**Conclusions:**

We present a pilot study for transcriptome involvement in coronary artery calcification and demonstrate how RNA-Seq analyses using LCLs as a tissue surrogate may yield fruitful results in a clinical sequencing project. In addition to canonical gene expression, we present candidate variants from alternative splicing and novel transcript detection, which have been unexplored in the context of this disease.

## Background

Coronary Artery Disease (CAD) is a leading cause of mortality worldwide. The etiology of CAD is influenced by lifestyle, genetics, and gut microbiome [1,2] although the balance of these contributors remains unclear. While numerous GWAS projects have explored the genetics of CAD susceptibility [3], comparatively less is known about gene expression changes in this disease [4]. This is partly because CAD is a complex phenotype involving multiple physiological systems. Consequently, no single tissue may fully reflect the network of gene expression changes underlying the disease [5].

In addition, current knowledge of the CAD transcriptome is based on gene expression microarrays, a technology that is useful but has several limitations. Recently, RNA-Seq, a powerful new technique for transcriptome analysis has revolutionized gene expression analyses by providing the ability to simultaneously interrogate all transcripts in an RNA sample (unlike microarrays which are limited to previously annotated transcripts) [6]. In addition, the high resolution of RNA-Seq can facilitate discoveries of transcript dysregulation that have been missed by previous technologies. Here, we present results from a pilot application of RNA-Seq on human cases and controls chosen to reflect extremes for coronary artery calcification (CAC), a clinical marker for advanced CAD that is highly correlated with future adverse cardiovascular events [7]. As a patient surrogate for gene expression, we used Epstein-Barr virus transformed lymphoblastoid cell lines (LCLs), which have been shown in multiple studies to reliably reflect gene expression signatures [8], particularly those associated with nearby *cis*-acting genomic polymorphisms (expression quantitative trait loci or eQTLs) [9].

Here, we used RNA-Seq to compare LCLs from matched CAC cases and controls in the ClinSeq® project [10], and report a set of candidate genes for CAD that show differential expression and splicing variation between the groups, as well as novel transcripts that may have relevance for disease. RNA-Seq results were experimentally and technically replicated by repeating the cell culture part of our experiments and then measuring gene expression independently using expression microarrays. At the network level, we demonstrate that these differentially expressed genes are enriched for prior CAD association as well as for biological pathways with direct relevance to atherosclerosis.

## Methods

An expanded Methods section is provided in Additional file 1.

### Coronary calcification scoring

CAC scoring was performed using multi-slice computed tomography (Aquilion One, Toshiba Medical Systems, Japan or Lightspeed VCT, General Electric Healthcare, Waukesha, Wisconsin) with the use of prospective electrocardiographic gating. Calcification was quantified using the Agatston scoring method [11] on a dedicated workstation (VitreaFX or GE Advantage). The study was approved by the NHGRI Institutional Review Board and all subjects gave written informed consent.

### Cell culture, RNA-Seq library preparation, and sequencing

LCLs were established by Epstein-Barr Virus (EBV) inoculation of B-lymphocytes using standard procedures, grown to a density of 2–3 × 10^5^ cells/ml, harvested when the culture reached 10^7^ cells in total (5–6 passages) and stored at -80°C. Total RNA from 4 × 10^6^ cells was extracted using the RNeasy Mini kit (Qiagen). RNA integrity was assessed using an Agilent Bioanalyzer (Agilent). RNA-Seq libraries were constructed using a custom protocol (see Additional file 1). Sequencing was done on Illumina GAII_x_ sequencers (Illumina), collecting two lanes of 51 bp reads for each library.

### RNA-Seq data processing

Reads were aligned to the human genome (hg18) using TopHat [12]. The TopHat output SAM file was converted to BED format using the bamToBed script from the BEDTools package [13]. The coverageBed script from BEDTools was used to count reads mapping to individual exons in the RefSeq database (see Additional file 1). A total count for each transcript in this database was obtained by adding the counts for its constituent exons. These counts, after filtering and normalization (see Additional file 1), were used for statistical analysis of differential expression. We confirmed the subject identity in all RNA-Seq libraries by genotyping a subset of expressed SNPs from the corresponding genomic DNA. This indicated some contamination in the data for subject 133871; hence, this subject was excluded from further RNA-Seq analyses. The transcript-level RNA-Seq counts were first analyzed using a two level, one-way ANOVA to compare the expression in the case and control groups. Concurrently, the count data were analyzed using the edgeR Bioconductor package with default settings using the moderated tagwise dispersion option and the prior. N parameter set to 4.

### Affymetrix exon array experiment

For each sample, two micrograms of total RNA were used in conjunction with the Whole-Transcript Expression Analysis protocol for Affymetrix Human Exon 1.0 ST arrays. Arrays were scanned using an Affymetrix Gene Chip Scanner 3000 and intensities were calculated using Affymetrix AGCC software. RMA (Robust Multichip Average) signal intensities were calculated using Affymetrix Expression Console, converted to arithmetic scale, and transformed using an adaptive variance-stabilizing, quantile-normalizing transformation that was scaled to match the transformation used on the RNA-Seq data. A two level, one-way ANOVA was run on the microarray expression results.

### Western blot analysis of IGLL5

A small piece of human carotid atherosclerotic plaque was frozen in liquid nitrogen and crushed to fine powder using an alloy tool steel mortar and pestle set that was pre-cooled in liquid nitrogen. The tissue powder was transferred to an ice-cold microcentrifuge tube and ice-cold cell lysis buffer (RayBiotech #0103004-L) containing protease inhibitor cocktail (Sigma P8430) was added; the tissue weight to buffer volume ratio was 0.1 g/0.3 ml. The samples were vortexed for 20 seconds at 10-minute intervals for 30 mins (and were kept on ice when not being vortexed). Samples were then centrifuged at 14000 g for 15 minutes at 4°C; the supernatant was collected in new microcentrifuge tubes. Protein concentrations in the supernatant were quantified by using Micro BCA Protein Assay Kit (Pierce #23225) according to manufacturer’s instructions. 50ug of protein from each sample was separated on a 4 – 20% polyacrylamide gel (Bio-Rad, #161-1159) with Tris-glycine – SDS buffer (Bio-Rad, #161-0732). The proteins were transferred onto Immuno-Blot PVDF membranes (Bio-Rad, #162-0174). The membrane was blocked with 5% non-fat dry milk in Tris-buffered saline Tween 20 (0.5%) for 1 hour at room temperature, then incubated with primary antibody (anti human IGLL5, Abgent #AP18459b) at a concentration of 1:200 overnight at 4°C. The membranes were washed with Tris-buffered saline Tween 20 (0.5%) three times, incubated with a secondary peroxidase – linked antibody (anti rabbit IgG HRP conjugate, Bio-Rad #170-6515) for two hours at room temperature and then washed three times with Tris-buffered saline Tween 20 (0.5%). The reactive bands were visualized by a chemiluminescence kit (Bio-Rad, #170-5040) on Kodak BioMax Light film. The intensity of each IGLL5 band was analyzed by using the Chemidoc XRS system with Western blot analysis software (Bio-Rad). The final intensity was adjusted by individual loading control intensity readings (beta-actin).

### Detection of novel transcripts

The Ensembl, AceView, ccDs, knownGenes, refGene, tRNA and rnaGene annotation tables were downloaded in GTF format from the UCSC Genome Browser (http://www.genome.ucsc.edu) and combined into one master annotation file using the Cuffmerge tool in Cufflinks [14]. Transcripts were assembled using RNA-Seq data from a separate LCL library with extremely high depth of sequencing (~200 million read pairs) using Cufflinks with the -M option to mask transcripts in the combined annotation file. These putative novel transcripts were then used as a reference annotation to run Cuffdiff, with the same BAM files from the case and control subjects that were used for quantifying gene expression. Loci detected as significant after Benjamini-Hochberg multiple testing correction were visually inspected by viewing the raw sequencing data (in BAM and BigWig format) on the UCSC Genome Browser with all gene and gene prediction tracks (and the ENCODE gene regulation track) turned on.

### Detection of alternatively spliced transcripts

For alternative splicing, the exon-level counts were fit to a linear mixed-effect three-factor ANOVA model containing the following terms:

$$\begin{array}{l} y_{\mathrm{ijk}}=\mu +A_{i}+\beta_{j\left(i\right)}+C+AC_{\mathrm{ik}}+\epsilon_{\mathrm{ijk}} \end{array}$$

where *y* was the normalized read count for an exon, *A*_*i*_ was the fixed treatment effect for 1 through *i* treatments (in this case, the case or control status), *β*_*j* (*i*)_ was the random sample effect for sample *j* within treatment, *C*_*k*_ was the fixed exon effect for 1 through *k* exons within a transcript, *AC*_*ik*_ was the fixed interaction “treatment X exon” effect and ϵ was the error factor. The ANOVA p-value for p-*AC* (which indicates the strength of the exon-treatment interaction) was then used to select for exons showing significantly different usage between cases and controls. Independently, the cuffdiff algorithm [14] was also used to detect alternatively spliced transcripts.

## Results

### Assessment of CAD burden and RNA-Seq experimental design

To quantify CAD status, study subjects were assessed at enrolment for CAC, using multi-slice computed tomography followed by Agatston scoring (see Methods). CAC scoring is a robust marker of CAD [15] and has been demonstrated to be useful for both calibration and discrimination of the disease burden. In addition, this measurement of CAD also has superior positive predictive value for future adverse cardiovascular events [16,17]. We selected eight age-, sex-, and ethnicity-matched case:control pairs from the extremes of the coronary calcium score distribution for combined RNA-Seq and microarray analysis and another eight matched case:control pairs for microarray-only analysis (Table 1). The median CAC scores for cases in the first and second groups were 1531.5 and 682.5, respectively. For comparison, even the presence of a CAC score (i.e., any non-zero value) is clinically considered indicative of CAD, while a score of 400 is often considered an advanced disease state [17]. When the median age for the cases in the first and second groups (56 and 61.5 years, respectively) and the ethnicity of these subjects (Caucasian) was considered, these scores corresponded to the 99^th^ and 93^rd^ centiles, respectively, as measured using the CAC score distribution from 6110 participants in the Multi-Ethnic Study of Atherosclerosis (MESA) [18]. This demonstrated the severity of CAD in our discovery cases. As part of the ClinSeq® protocol, all subjects were also analyzed using a set of 123 clinical chemistry tests and six phenotypic measurements (Additional file 2: Table S1). Results from these tests did not show significant association with calcification scores.

**Table 1:** Clinical data for 32 subjects

**Controls**			**Cases**		
**ID**	**age**	**Calcification score**	**ID**	**age**	**Calcification score**
**Discovery cohort (RNA**-**Seq** **+** **Microarray)**					
**112916**	56	0	**163383**	55	1114
**116099**	62	0	**108971**	63	1370
**119657**	53	0	**198564**	53	1012
**133237**	53	0	**121424**	52	514
**113235**	53	0	**165583**	53	4352
**118655**	58	0	**189793**	57	3532
**119522**	65	0	**158592**	66	2885
**133871**	56	0	**132306**	59	1693
**Validation cohort (Microarray)**					
**199039**	64	0	**129854**	64	1268
**124644**	58	0	**106945**	58	890
**199794**	58	0	**116106**	58	697
**102488**	63	0	**108828**	64	693
**184917**	57	0	**155489**	57	672
**154405**	60	0	**148238**	61	668
**117532**	62	0	**142361**	63	654
**192736**	62	0	**107143**	62	565

All subjects were males of Caucasian ethnicity.

### Analyses of gene expression with RNA-Seq data

**Figure 1:**
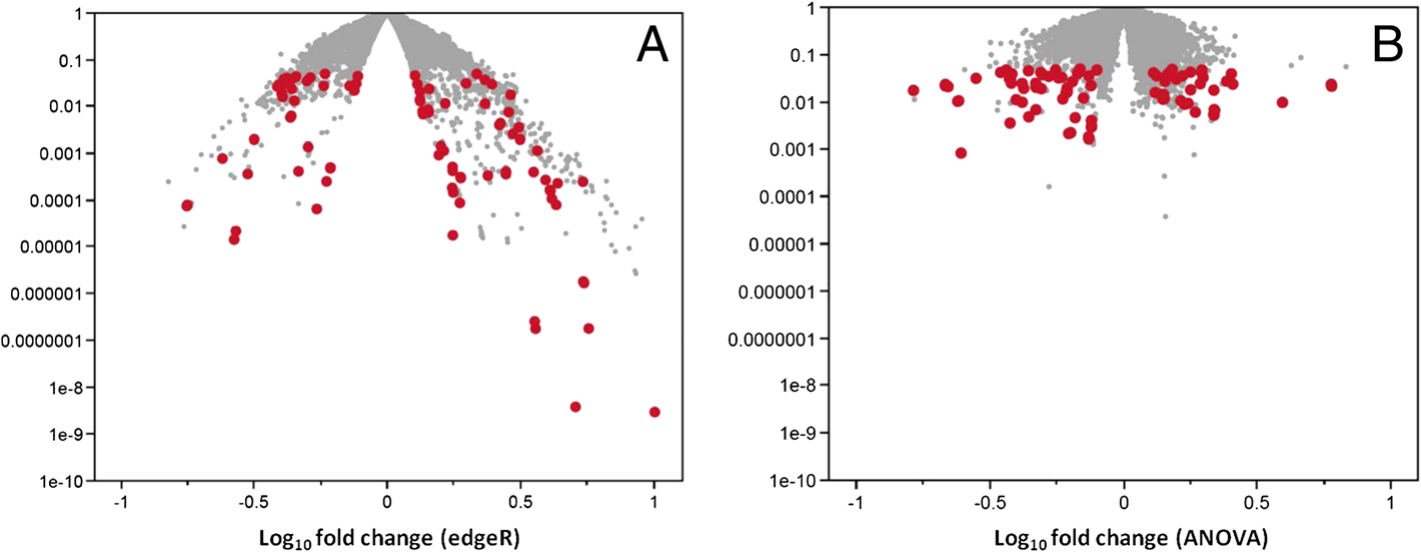
**Volcano plots from edgeR (panel A) and ANOVA (panel B) analyses of RNA**-**Seq count data.** X-axis on both panels shows base 10 logarithm of fold change (case/control). Y axis shows p value. Red dots indicate 186 transcripts meeting p < 0.05 in both tests.

To convert the RNA-Seq read data into a quantitative measure of gene expression, we calculated the number of RNA-Seq reads mapping to transcripts in the RefSeq database (see Methods). Such transcript-level read counts are better suited for statistical testing of gene expression differences compared to other RNA-Seq expression metrics [19]. Several statistical techniques have been developed for quantifying differential expression from RNA-Seq count data. To reduce artifacts arising from an arbitrary choice of any single technique, we used a dual analysis approach. First, we analyzed the count data with the Bioconductor package *edgeR*[20], which uses the negative binomial distribution to model the count data. Using the common dispersion setting in *edgeR*, we found 978 transcripts to be differentially expressed transcripts (p < 0.05), of which 55 were significant after applying a 5% Benjamini-Hochberg correction for multiple testing (Figure 1A). However, *edgeR* and other RNA-Seq analysis tools using the negative binomial distribution have high rates of false discovery [21]. To reduce artifacts arising from an arbitrary choice of any single technique, we applied a second, more conservative test (one-way ANOVA) (Figure 1B) in addition to *edgeR*, which returned 515 transcripts at p < 0.05 without correction for multiple testing. Overall, results from both techniques showed good agreement, although edgeR was more liberal than the ANOVA test in assigning low significance values. To define a gene list, we selected transcripts that were significant at p < 0.05 in both the edgeR and ANOVA results, retaining 186 transcripts from 116 genes (Additional file 2: Table S2). We noted here that neither edgeR nor ANOVA tests were designed for isoform-level quantification; hence, for genes with multiple transcripts in this list of 186, we could not identify which isoform was differentially expressed.

**Figure 2:**
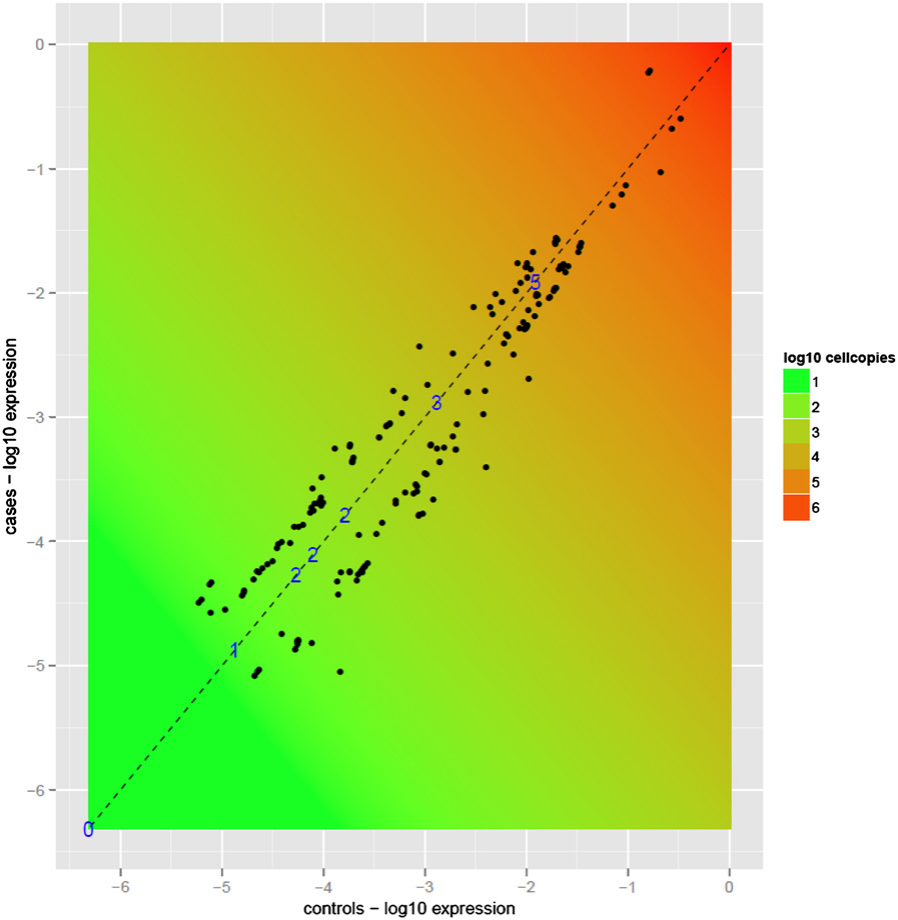
**Plot showing RPKM values of 186 differentially expressed transcripts and their absolute expression (measured as copies/cell).** Log10 RPKM values for controls and cases are shown on the x and y-axes, respectively. The dashed diagonal line represents equal RPKM values for both cases and controls, and hence no differential expression. Thus, the orthogonal distance to the dashed line of a point indicates the amount of differential expression. The blue numbers on the dashed line indicate the log10 measure of copies/cell for the seven spike-in RNA controls (ranging from 1–100000), and the position along the x and y-axes indicate the corresponding RPKM values recorded for these spikes. Based on a linear regression model for copies/cell as a function of RPKM, predictions for absolute expression measured as copies/cell for the 186 transcripts were made from these spike-in values. For each position, the average of the RPKMs corresponding to the x and y-coordinate values yield predictions of cell copies based on this model and a corresponding color shown in the background of the plot ranging from green (low) to red (high).

An interesting feature of the RNA-Seq results was that many transcripts expressed at very low levels showed significant differences between cases and controls. As genes expressed at near-background levels have historically been problematic for microarray technology [22], this aspect of the CAD transcriptome may benefit greatly from the increased dynamic range of RNA-Seq. To quantify absolute expression of these rare transcripts, we conducted a separate RNA-Seq experiment, starting with the same amount of total RNA used for other experiments in this study, with the difference being that the number of cells used for RNA extraction was precisely quantified. To the input RNA, we added seven polyadenylated non-mammalian RNA spikes, quantified such that their abundance (measured as copies per cell) spanned five orders of magnitude (Additional file 2: Table S3). Next, after sequencing this library to the same depth as the others (~40 million reads), we converted RNA-Seq reads counts of these spikes to RPKM (Reads Per Million per Kilobase) values [19]. Finally, by comparing RPKM values of differentially expressed transcripts in our study with the spike-in control experiment, we determined that the cellular abundance of the rarer transcripts in our data approaches ~5 copies per cell (Figure 2). As many of the candidate genes with the highest statistical significance belong to this low-expression category (including *LOC100131347* which had the lowest p value in our ANOVA results), some observations are relevant to note here. Firstly, before statistical testing, we screened out transcripts which had minimal or no coverage (see Additional file 1). Hence, these results are unlikely to represent statistical artifacts arising from low RNA-Seq coverage. Regarding quantity of input material, ten micrograms of total RNA were used for all RNA-Seq experiments, which is far above the minimum requirement for the Illumina RNA-Seq protocol. Consequently, paucity of input RNA should not cause transcripts to appear “rare” if they are otherwise expressed abundantly. Finally, each sample was sequenced using two full lanes of an Illumina flow cell for a relatively high depth of 40 million reads, which suggests that lack of sequencing coverage would not bias our results. Overall, these findings suggest that low-expression transcripts (many of which may have escaped detection with previous techniques) constitute an unexplored area of the CAD transcriptome that may contain clinically relevant information.

### Validation of RNA-Seq results using affymetrix arrays

To address the effects of variation introduced by experimental procedures, we repeated the cell culture and RNA extraction steps for all 16 cell lines used for the RNA-Seq, starting from previously frozen stocks. We then measured gene expression in these newer cultures using Affymetrix Human Exon 1.0 ST microarrays. Previous studies have demonstrated that these two methods of expression measurement (RNA-Seq and microarray) show strong agreement when applied to the same RNA sample [23]. Here, despite the fact that two additional passages of the cell lines took place in between the separate experiments, comparisons of the sixteen pairs of RNA-Seq and microarray results from the same subjects still showed high correlation (average R = 0.73, p < 0.001) (Additional file 1: Figure S 1). Hence, we conclude that *in vitro* procedures did not appear to cause significant variability in the gene expression profiles of the cell lines.

**Figure 3:**
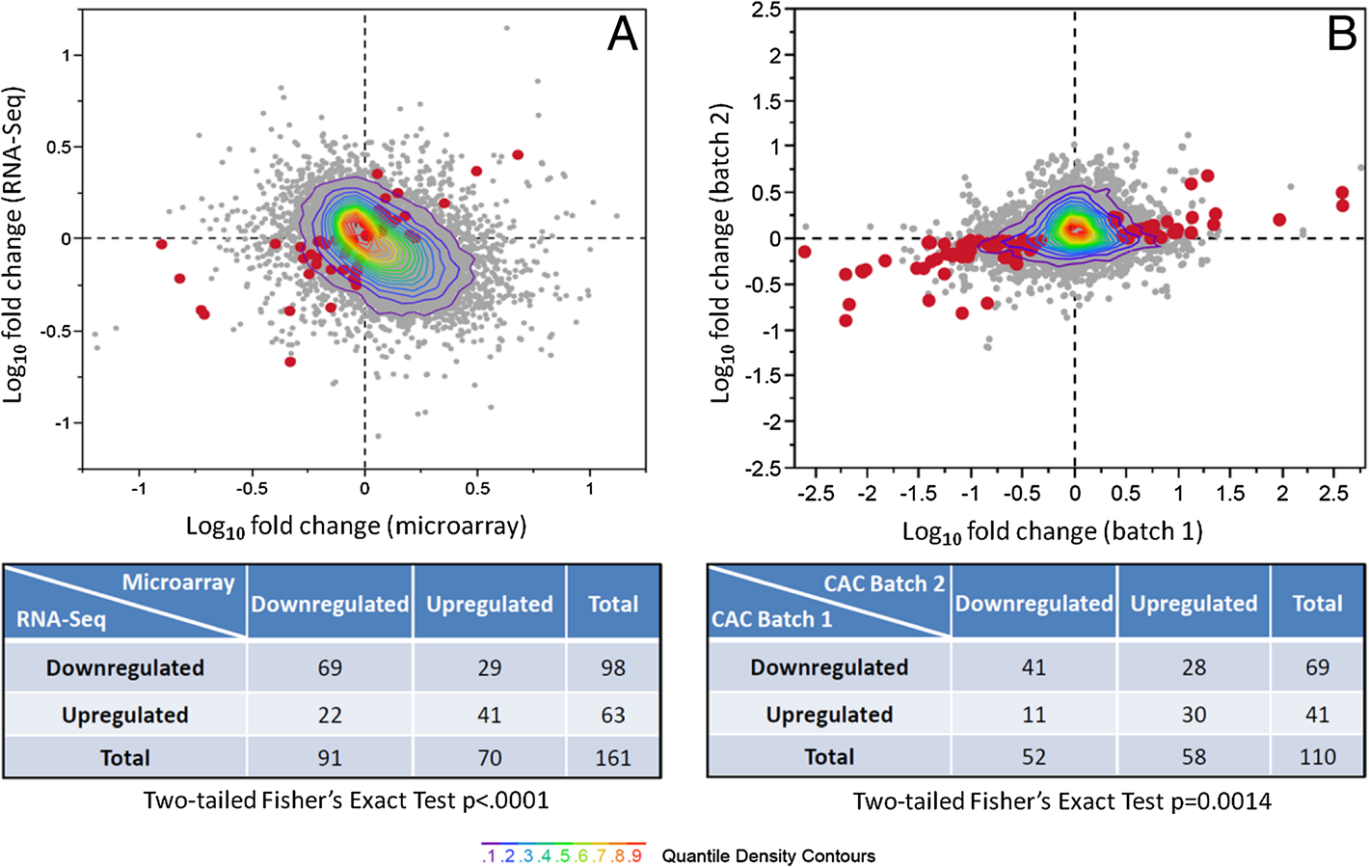
**Validation of RNA**-**Seq results with microarray data. (A)** Fold changes (case/control) from first 16 subjects, measured by RNA-Seq (Y-axis) and microarrays (X-axis) in logarithm base 10 scale. Red dots show 110 transcripts (out of 186 in Figure 1) that were upregulated or downregulated in both experiments. **(B)** Comparison of microarray data from first and second groups of 16 subjects (X- and Y-axes, respectively). Red dots show 71 transcripts out of 110 in panel A that were upregulated or downregulated in both groups. Contingency tables for statistical calculations are shown below each panel.

To identify gene expression differences in cases and controls that were robust to both experimental (i.e., cell culture) replication as well as measurement technique, (i.e., RNA-Seq or microarray), we next selected genes that showed consistent up- or downregulation in both experiments. From the list of 186 differentially expressed transcripts found by RNA-Seq, 161 were included in the microarray probe design. Of these, 110 were regulated in the same direction (i.e. showed consistent upregulation or downregulation) in both the microarray and RNA-Seq data (Figure 3A). Given that these two gene expression measurements were conducted independently and on different RNA extraction batches, the observed agreement in fold change for 110/161 genes is highly significant (one-tailed Fisher’s Exact Test, p < 0.0001; Additional file 2: Table S4). Next, we selected a validation cohort of eight additional matched case:control pairs from the extremes of the CAC score distribution (Table 1) and analyzed these samples using Affymetrix Human Exon 1.0 ST microarrays. Despite the fact that cases in this cohort had a lower median calcification score compared to the first cohort (682.5 and 1521.5, respectively), we again found a significant number of transcripts (71 of the above 110) with consistent up- or downregulation in both cohorts (one-tailed Fisher’s Exact Test, p = 0.0014; Figure 3B; Additional file 2: Table S5). We conclude that these transcripts may be potential CAC biomarkers, as their expression differences persist even though the extent of calcification differed substantially when comparing our first and second cohorts.

**Figure 4:**
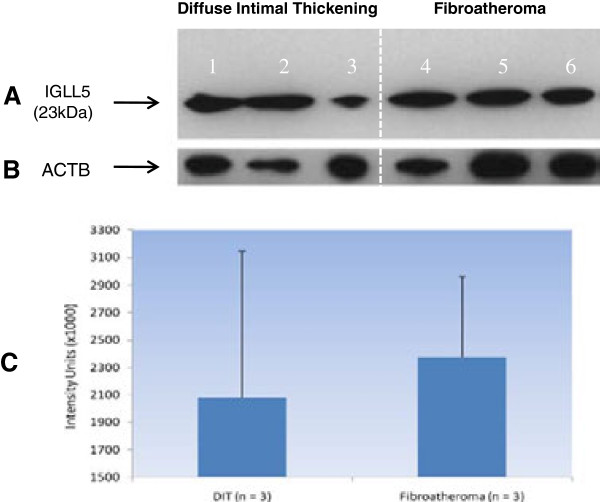
**Immunoblot demonstrating the expression of IGLL5 in carotid plaques obtained at endarterectomy.** Lanes 1–3: lysates from an area exhibiting diffuse intimal thickening (DIT). Lanes 4–6: matched lysates of fibroatheromatous plaque from the same samples. Panel **A**: anti-human IGLL5 antibody staining. Panel **B**: Beta-actin band used for densitometric quantification of IGLL5. Panel **C**: Increased trends of IGLL5 expression in fibroatheromatous plaques. See also Additional file 1: Figure S3 for siRNA knockdown experiments of to establish specificity of this antibody for IGLL5.

Finally, we reasoned that direct evidence of a causal relationship with CAD would be obtained if any transcripts from LCL-based analyses exhibited differential expression patterns within actual atherosclerotic lesions. To identify such genes, we downloaded publicly available microarray data (GEO accession number GSE28829) from a gene expression study of human carotid artery autopsy samples [24]. From these samples (which were part of the Maastricht Pathology Tissue Collection), RNA was collected from 13 arteries in early CAD states (pathological intimal thickening and intimal xanthoma) and from 16 advanced CAD lesions (thin or thick fibrous cap atheroma). The two groups of samples, which were clearly separated by Principal Components Analysis, showed an abundance of genes with highly significant differential expression (4039 genes with p < 0.05, one-way ANOVA test with 10% Benjamini-Hochberg FDR correction, Additional file 1: Figure S2). However, our aim here was not to study these samples themselves but to identify genes showing similar patterns in LCLs and atherosclerotic arteries. To identify such genes, we calculated fold changes of 20639 genes that were measured in both studies (i.e. RNA-Seq in our project and the Affymetrix U133 Plus 2.0 microarrays used for the autopsy RNA study). Next, we selected genes that had a fold change ≥ 1.25 in the same direction in all three data sets (i.e. first and second sets of CAC subjects in our study and in the CAD autopsy samples). Two genes (*MMP7* and *IGLL5*) met this criterion, of which, *MMP7* is especially notable due to its functional role in atherosclerotic plaque rupture and vascular smooth muscle cell apoptosis [25,26]. In addition, at the protein level, plasma concentrations of MMP7 were increased in patients with coronary artery disease [27]. Regarding *IGLL5*, this gene is interesting, as apart from being upregulated with disease in two unrelated tissues, it also had the largest fold change in the list of 186 significant transcripts. To investigate if *IGLL5* is successfully translated in the arterial wall and if the mRNA difference persists at the protein level, we conducted preliminary immunoblotting experiments on lysates from human carotid plaques. Using an anti-human polyclonal antibody (which we confirmed to be IGLL5-specific using siRNA knockdown experiments; Additional file 1: Figure S3A-B), we compared three samples each from areas exhibiting early and advanced CAD (diffuse intimal thickening and fibroatheroma, respectively). Corresponding to the mRNA upregulation, we found a trend of increased protein levels in lysates from advanced disease (Figure 4). IGLL5 was also detected at abundant levels in the supernatants of the above lysates, suggesting that it is a secreted protein (Additional file 1: Figure S3C-D). Although the trend of protein upregulation was not statistically significant in this small set of samples (Additional file 2: Table S6), given the complexity of CAD etiology, follow-up studies of this gene using a larger sample size may be worthwhile.

### Analyses of transcription at unannotated genomic regions

**Figure 5:**
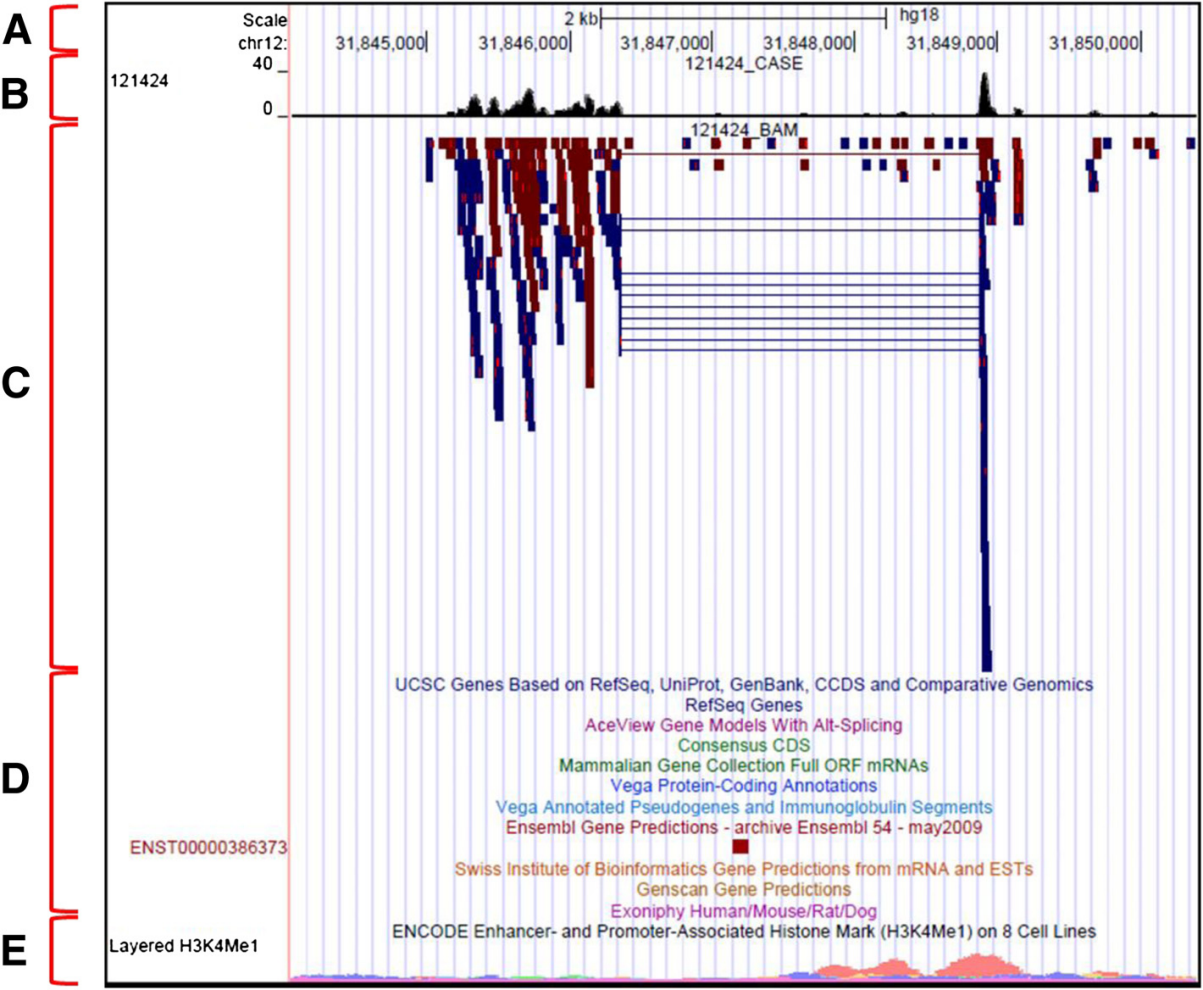
**Putative novel transcript on chromosome 12 detected by Cufflinks.** UCSC Genome Browser tracks in vertical order: **(A)** Chromosomal location. **(B)** WIG file showing expression levels. **(C)** BAM file showing mapped RNA-Seq reads (blue bars for forward strand; red for reverse). Segments joined by thin lines represent reads spanning a putative splice junction. **(D)** Assorted gene and gene prediction tracks showing absence of prior annotations for the novel transcript. **(E)** ENCODE enhancer- and promoter-associated histone mark H3K4Me1.

Recent evidence from RNA-Seq studies has demonstrated that the human genome is pervasively transcribed [28]. Thus, it is possible that CAD-relevant expression differences may exist in unannotated parts of the genome. Such differences would be even more interesting, as previous gene expression studies of CAD using microarrays would have entirely missed these regions due to their absence in the probe design. To explore this aspect of the transcriptome, we used the Cufflinks suite of RNA-Seq analysis tools (see Methods) [14]. First, we assembled novel transcripts, which we defined as those not included in a combination of several previous annotations including predicted genes. Next, we used the Cuffdiff tool to measure differential expression in these novel transcripts. Finally, we manually inspected the results to confirm that they showed evidence for being putative transcripts. Using this approach, we found eight novel loci that show differential expression with high statistical significance (p < 0.0001 after Benjamini-Hochberg correction) (Table 2). In terms of transcript structure, five of these eight loci contain multiple regions of increased read coverage joined by spliced RNA-Seq reads that mapped following the GT-AG rule (Figure 5). This strongly suggested the presence of a canonical exon-intron structure. Mapping these loci to the ENCODE Integrated Regulation track on the UCSC Genome Browser showed histone modifications indicating active transcription. Although functional analyses will be required to confirm if these transcripts have genuine relevance to CAD, these results are interesting as they form the first description of unannotated regions of the transcriptome associated with this disease. As the ability to discover such novel transcripts is a significant advantage of RNA-Seq over existing techniques, we speculate that future analyses in different tissues and with larger sample sizes will continue to demonstrate that hidden components of CAD biology may exist outside known genes.

**Table 2:** **Novel transcripts detected by Cufflinks**/**Cuffmerge**/**CuffDiff pipeline**

**Name**	**Location**	**Fold change (case/control)**	**Corrected p value**	**Spliced?**	**Histone mark?**	**EST?**
CUFF.11349	chr8:101524720-101588587	-1.88185	0.041685	Yes	Yes	Yes
CUFF.2472	chr12:31838565-31853772	-2.26342	0.123977	Yes	Yes	Yes
CUFF.3	chr1:80691-80824	-11.5273	4.46E-08	No	Yes	Yes
CUFF.4	chr1:452495-461228	-10.6557	7.54E-10	Yes	Yes	Yes
CUFF.5*	chr1:163653-164729	-12.5567	1.96E-10	No	No	Yes
CUFF.6*	chr1:164811-166159	-12.8975	1.27E-12	No	No	No
CUFF.7*	chr1:166235-166948	-12.7567	1.27E-12	Yes	No	Yes
CUFF.9	chr1:227665-257075	-11.1513	1.18E-08	Yes	Yes	Yes

Column 5 denotes presence of spliced reads following GT-AG rule. Column 6 denotes presence of layered H3K4Me1 or H3K4Me3 histone modification marks at the locus. Column 7 denotes the presence of spliced or unspliced human ESTs in the UCSC Genome Browser database. *CUFF.5, CUFF.6 and CUFF.7 were annotated by the Cufflinks pipeline as separate transcripts but their close location suggests that they may be components of the same transcript.

### Detection of candidate genes for alternative splicing

**Figure 6:**
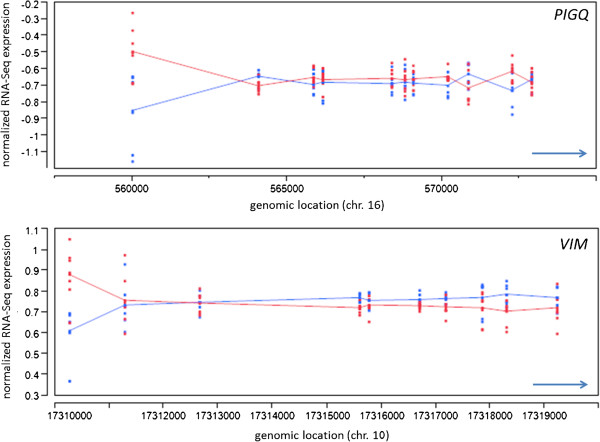
**Examples of alternative splicing detected using exon**-**level analysis of RNA-Seq counts in *PIGQ *(top panel) and *VIM *(bottom panel).** X axis shows genomic location of exons (red and black dots for cases and controls, respectively) in hg18 coordinates. Y axis shows mean-subtracted normalized RNA-Seq read counts for each exon. Arrows at bottom right show direction of transcription. Neither gene was differentially expressed when all exons were considered together.

The role of alternative splicing in CAD overall (or CAC in particular) has not been explored comprehensively, mainly due to the difficulties of microarray platforms in efficiently detecting changes in splicing patterns. In contrast, RNA-Seq is extremely powerful for studying splicing variation. As statistical algorithms for such analyses are in an early stage of development, we chose to proceed with a conservative approach for detecting case:control splicing changes. Specifically, we analyzed exon-level RNA-Seq read counts using a linear ANOVA model fit to exon-level RNA-Seq read counts (see Methods). This model was designed to detect genes containing exons with increased case:control differential expression relative to other exons within the same transcript , which would thus be potential candidates for exon skipping, the most common form of alternative splicing. Using this approach, we detected 45 transcripts that show statistically significant differential usage in at least one exon (p*AC* < 0.05 after 10% FDR correction) (Figure 6). These transcripts were then validated independently using the cuffdiff computational algorithm [14]. Eleven transcripts were validated by both methods and hence show promise for follow-up studies (Additional file 2: Tables S7 and S8). Interestingly, this list included the human leukocyte antigen gene *HLA*-*H*, variation in which has been associated with CAD susceptibility [29].

### Differentially expressed genes are enriched for CAD association and disease-relevant functions

We next determined if differentially expressed genes showed an overall relationship with cardiovascular disease. To investigate this, we first combined previously compiled lists of CAD-associated genes [30,31]. From the combined list, we identified 3424 genes which showed expression in our RNA-Seq data. From this list, 43 genes are present in the list of 186 differentially expressed transcripts in our results. Statistically, this represented a highly significant enrichment of CAD-associated genes (one-tailed Fisher’s Exact Test, p = 0.0002; Additional file 1: Figure S4), suggesting that gene expression differences we detected by comparing cell lines from CAC cases and controls showed an overall relationship with CAD.

At the network level, differentially expressed genes may identify biological pathways that are perturbed in CAD. To investigate this hypothesis, we used Ingenuity Pathways Analysis™ (IPA) software, which performs systems biology analysis using a large repository of previously documented gene-gene interactions and functional annotations (Additional file 3). The highest-scoring network (IPA score = 48) in the list of 186 differentially expressed transcripts included the term “Cardiovascular System Development and Function”, further highlighting the relationship of differential expression in this study with CAD. Interestingly, the second highest-scoring network (IPA score = 36) contained the function “Lipid Metabolism”, suggesting another possible mechanism through which gene expression differences may be related to CAD etiology. Finally, Gene Ontology (GO) term analysis of the 186 transcripts (using only LCL-expressed genes as background to avoid bias), detected a number of networks related to cyclic AMP metabolism (Additional file 2: Table S9).

## Discussion

We present here results from a pilot project for the use of RNA-Seq and cell lines in studying CAC gene expression. We used gene expression microarrays and cell culture replication for technical and experimental validation, respectively, of our initial results from this new technology. This project should be viewed in context of its scope and limitations; analyses of much larger numbers of LCLs will be required before any definitive statements can be made about the functional role in CAC of genes that we detected. However, our primary objective in this study was not functional analysis of such genes, but rather to identify candidate dysregulated genes in CAC using two relatively unexplored tools that may be useful overall in CAD research. By choosing LCLs from subjects who represent extreme outliers for the calcification score distribution, we hypothesized that between-group differences that are relevant for CAD may be detected. Evidence for this is reflected in the significant enrichment of CAD-associated genes in our results and further underscored in our IPA results, which show that differentially expressed genes we detected are concentrated in functional networks directly connected to CAD. However, we do not suggest that LCLs mirror in any way the transcriptional landscape of the atherosclerotic lesion itself. Rather, we hypothesize that our results represent eQTL-associated gene expression variants, which are stably propagated in these cell lines, underlying the observed differences between the case and control groups. We show that the combination of RNA-Seq and microarrays can be used to obtain useful results with a comparatively small sample size. In the near future, we anticipate that rapidly falling sequencing costs and development of user-friendly data analysis tools will soon make RNA-Seq the preferred alternative for transcriptome studies.

As the scope of this pilot project did not include functional validation assays, we refrain from speculating on the specific mechanisms by which individual genes may be involved in atherosclerosis development. However, as to the best of our knowledge, this study represents the first whole-transcriptome analysis specifically focusing on coronary artery calcification, and differentially genes that we found include many previous CAD candidates (such as *MMP7*, *EPAS1*, *ESAM*, *CASP1*, *GUCY1A3*, *CLCN4*, *LEF1*, *ENPP5*, and *ZHX2*), some observations may be relevant to state here. Interestingly, we detected differential expression of *ZHX2*, a regulator of plasma lipid metabolism that is the closest gene to the top SNP locus from a recent atherosclerosis GWAS meta-analysis [32]. At the gene family level, the *GIMAP* family is enriched, with four genes (*GIMAP1*, *GIMAP4*, *GIMAP5*, and *GIMAP7*). Perhaps not by coincidence, multiple *GIMAP* family members have also been reported in a set of 128 genes (the “A-module”) that is associated with atherosclerosis development. Overall, the *GIMAP* family has a crucial role in the development and function of T lymphocytes, which have an important role in the etiology of atherosclerosis [33]. Finally, it is intriguing that we detected differential expression of four genes involved in cAMP metabolism (*ADM*, *APLP1*, *PRKCA* and *PTHLH*), as existing studies focusing on individual genes involved in arterial calcification collectively suggest that perturbation of ATP metabolism plays a role in this process [34].

## Conclusions

In summary, in this project we piloted the study of coronary artery calcification using cell lines as a patient surrogate for gene expression. We demonstrate that with careful experimental design and secondary validation of results, statistically significant results can be obtained with RNA-Seq even from a small sample size. In addition to canonical gene expression, we focused on alternative splicing and novel transcript discovery, two areas of the CAD transcriptome that may benefit the most from further RNA-Seq analyses. Studies using larger numbers of LCLs along with follow-up experiments will be needed to validate differentially expressed genes from this pilot study as true CAC biomarkers; this will become possible in the near future as we deploy RNA-Seq to the entire ClinSeq® cohort.

## Abbreviations

LCL: Lymphoblastoid cell line; EBV: Epstein-Barr virus; CAD: Coronary artery disease; CAC: Coronary artery calcification; GWAS: Genome-wide association study; FDR: False discovery rate.

## Competing interests

Leslie G. Biesecker is an uncompensated consultant to the Illumina Corporation.

## Authors’ contributions

SKS, LGB, JCM, EDG and PJM designed the experiments. MYC and AEA carried out all CT scanning and calcification scoring procedures. SL performed all cell culture experiments. SKS, DSA and NISC constructed and sequenced RNA-Seq libraries. AGE performed all microarray experiments. FDK, XZ and QC conducted all immunoblotting experiments. SKS, JJB, PFC, LNS, JCM and PJM participated in bioinformatics and statistical analysis of the RNA-Seq and microarray data. SKS and JJB drafted the manuscript. All authors read and approved the final manuscript.

## Supplementary Material

Additional file 1 **Additional Methods and Figures Description: Expanded RNA-Seq protocol, statistical and bioinformatics methods.** Additional file 1: Figures S1–S4. Click here for file

Additional file 2 **Additional Data.** Description: Clinical data, expanded lists of differentially expressed and alternatively spliced transcripts and results from Western blotting. Click here for file

Additional file 3 Ingenuity Pathways Analysis results Description: Complete output from Ingenuity Pathways Analysis. Click here for file
