# Supplementary material for: Identification of candidate genes involved in coronary artery calcification by transcriptome sequencing of cell lines
Source: BMC Genomics. 2014 Mar 14;15:198. doi: 10.1186/1471-2164-15-198 (PMC4003819; doi:10.1186/1471-2164-15-198)
Supplement: Additional file 1 — Additional Methods and Figures Description: Expanded RNA-Seq protocol, statistical and bioinformatics methods. Additional file 1: Figures S1–S4. [file 1471-2164-15-198-S1.docx]

ADDITIONAL MATERIAL

**Contents**

1. Additional Methods (with Appendices A and B)
2. Additional Figure 1: Correlation between RNA-Seq and microarray results
3. Additional Figure 2: ANOVA results from carotid artery Affymetrix arrays
4. Additional Figure 3: siRNA knockdown experiments for IGLL5 antibody
5. Additional Figure 4: Fisher’s Exact Test results for CAD association enrichment
6. Additional References

**Detailed methods**

CT scanning and assessment of calcification score

Coronary calcium scoring was performed using multi-slice computed tomography (Aquilion One, Toshiba Medical Systems, Japan or Lightspeed VCT, General Electric Healthcare, Waukesha, Wisconsin) with the use of prospective electrocardiographic gating. Coronary calcium was quantified using the Agatston scoring method[[1](#_ENREF_1)] on a dedicated workstation (VitreaFX, Vital Images, Minnetonka, Minnesota or GE Advantage, General Electric Healthcare, Waukesha, Wisconsin). Matched case-control pairs were chosen based on the extremes of the coronary calcification score distribution. All testing was approved by the NHGRI Institutional Review Board and all subjects gave informed consent for whole-genome and whole-transcriptome sequencing at the time of recruitment.

Cell culture (EBV transformation/growing cell lines)

LCLs were established by Epstein-Barr Virus (EBV) inoculation of B-lymphocytes using standard procedures [[2](#_ENREF_2)]. Cultures were seeded with 10^6^ cells in 5 ml of RMPI1640 (Invitrogen, CA) with 20% Fetal Bovine Serum (Invitrogen, CA), incubated at 37^0^ C with 5% CO_2_ and grown at a density of 2-3x 10^5^ cells/ml. Cells were harvested when the culture reached 10^7^ cells in total and stored at -80^0^ C.

RNA-Seq library preparation and Illumina sequencing

Total RNA from aliquots of 4 x 10^6^ cells was extracted using the RNeasy Mini kit (Qiagen, MD). RNA integrity was assessed using an Agilent Bioanalyzer (Agilent, CA). RNA-Seq libraries were constructed using the protocol provided in Appendix A below (which is a modified version of the protocol used by Marioni et al (2008)[[3](#_ENREF_3)]. Sequencing was done on Illumina GAII_x_ sequencers (Illumina, CA), collecting two 51bp lanes of data, corresponding to ~40 million reads for each library.

RNA-Seq data processing

Illumina reads were aligned to the human genome (hg18) using TopHat (ver. 1.1.4)[[4](#_ENREF_4)]. The TopHat output SAM file was converted to BED format using the bamToBed script from the BedTools package [[5](#_ENREF_5)]. The coverageBed script from BedTools was used to count numbers of reads mapping to individual exons in the RefSeq gene annotation database. A total count for each transcript was obtained by adding the counts for its constituent exons. Detailed information about the TopHat and BedTools versions and settings used for this project is provided in Appendix B below.

Normalization of RNAseq data

Transcript-level counts were normalized by taking the column median across all transcripts for each subject, deriving the ratio of individual counts to this median value and by taking the base 10 logarithm of this ratio (which we term the LMed10 transform). This normalization technique is analogous to dividing by million mapped reads, (which is the mean mapped reads per gene * number of genes). However, rather than normalize by the mean, LMed10 normalizes by the median, which is less influenced by the few extremely highly expressed genes (globins, ribosomal RNA, mitochondrial RNA) sometimes observed.

Affymetrix probeset recoding to match RNA-Seq

Genomic coordinates of Affymetrix Human Exon 1.0 ST array probesets were mapped to RefSeq exon genomic coordinates. If more than one probeset mapped to the same exon, the average RMA value was used for the exon. This remapping procedure allows for a fair comparison of the RNA-Seq data and microarray expression levels for RefSeq transcripts.

Affymetrix Exon Array experiment

Samples were prepared according to Affymetrix protocols (Affymetrix, Inc). RNA quality and quantity was ensured using the Bioanalyzer (Agilent, CA) and NanoDrop (Thermo Scientific) respectively. Per RNA labeling, two micrograms of total RNA was used in conjunction with the Invitrogen RiboMinus protocol and the Affymetrix-recommended Whole-Transcript Expression Analysis protocol for the Human Exon 1.0 ST chips. An Affymetrix Gene Chip Scanner 3000 was used to scan the probe arrays and gene expression intensities were calculated using Affymetrix AGCC software. RMA signal intensities for “core” probesets were calculated using Affymetrix Expression Console (www.affymetrix.com). RMA values were exponentiated to the arithmetic scale, then quantile-normalized using an adaptive variance stabilizing transformation, termed S10[[6](#_ENREF_6)], which approximates the Log10 transformation used on the RNA-Seq count data.  The case-control differences of these transformed RMA values can be interpreted simply as Log10 fold changes, comparable to the Log10 fold changes computed on the RNA-Seq data.

IGLL5 siRNA knockdown experiments

5x10^5^ of frozen adult human negatively selected monocytes (Cat# 1009, Astarte Biologics, LLC, Redmond, WA) were seeded per well of 6-well plate in 2400 µL of Growth Medium containing RPMI1640 + 10% heat inactivated human AB serum + 1% Penicillin/Streptomycin (Cat# 11875127, 34005100 and 15070063. Life Technologies, Grand Island, NY). Cells were cultured at 37°C with 5% CO2 for 4 days. Shortly before siRNA assay, media was changed to 2300 µL of Macrophage Serum Free Media supplemented with 1% Penicillin/Streptomycin (Cat# 12065074. Life Technologies, Grand Island, NY). Fast-forward transfection with IGLL5 siRNA and GFP siRNA (Cat# SI05459657, SI05459664 and SI04380467, Qiagen, Valencia, CA) using HiPerFect Transfection Reagent (Cat# 301704, Qiagen, Valencia, CA) was performed according to manufacturer’s instruction. Wells without treatment or treated with GFP siRNA were served as controls. Briefly, 150ng of SI05459657, 300ng of SI05459664 and 150ng of SI04380467 were diluted in 100µL Macrophage Serum Free Media respectively. 12µL of HiPerFect Transfection Reagent was added to the diluted siRNA and mixed by vortexing. The mixtures were incubated for 10 minutes at room temperature and added drop-wise onto the cells. The plate was gently swirled to ensure uniform distribution of the transfection complexes and incubated at 37°C with 5% CO2 for 2 days. Supernatant was collected and cells were lysed for Western Blot.

**Appendix A**

**RNA-Seq library construction protocol**

**mRNA purification from Total RNA**

1. Dilute 10µg of total RNA with nuclease-free H_2_O to 100µL in a 1.5 mL RNase free non-sticky Eppendorf tube.
2. Heat the sample at 65°C for 5 minutes to disrupt the secondary structures, and place the tube on ice.
3. Meanwhile, aliquot 150µL of Dynal oligo(dT_25_) beads (Invitrogen #610-06) into a 1.5mL RNase free non-sticky eppendorf tube.
4. Wash the beads twice with 100µL of Binding Buffer (20mM Tris-HCl pH 7.5, 1.0M LiCl and 2mM EDTA), and remove the supernatant.
5. Resuspend the beads in 100µL of Binding Buffer, and add the 100µL of total RNA sample from step 2; rotate the tube at RT for 5 minutes, and remove the supernatant.
6. Wash the beads twice with 100µL of Washing Buffer B (10mM Tris-HCl PH 7.5, 0.15M LiCl, 1mM EDTA).
7. Prepare for second round of oligo-dT purification by aliquoting 80µL of Binding Buffer to a fresh 1.5mL RNase free non-sticky Eppendorf tube.
8. Remove the supernatant from the beads of step 6, add 20µL of 10mM Tris-HCl and heat the beads at 80^0^C for 2 minutes to elute mRNA. Immediately put the beads on the magnet stand and transfer the supernatant (mRNA) to the tube from step 7. Add 100µL of Washing Buffer B to the remaining beads.
9. Heat the mRNA sample from step 8 at 65°C for 5 minutes to disrupt the secondary structures, and place the tube on ice.
10. Meanwhile, wash the beads from step 8 twice with 100µL of Washing Buffer B, and remove the supernatant.
11. Add 100µL of mRNA sample from step 9 to the beads; rotate the tube at RT for 5 minutes.
12. Remove the supernatant and wash the beads twice with 100µL of Washing Buffer B.
13. Remove the supernatant from the beads, add 20µL of 10mM Tris-HCl and heat the beads at 80^0^C for 2 minutes to elute mRNA. Immediately put the beads on the magnet stand and transfer the supernatant (mRNA) to a fresh 200µL thin wall PCR tube, and there should be ~19µL of mRNA.
14. Add 100µL volume of DEPC-treated H_2_O.

**mRNA fragmentation on Covaris S2 AFA instrument**

1. Transfer mRNA to 6X16mm Covaris tube.
2. Fragment using the following settings: 10% duty cycle, 5 intensity, 200 cycles per burst, 60 seconds. Water bath temperature should not exceed 8°C.
3. Immediately transfer to a Microcon YM-30 column and spin for 3.5 mins at 10000 rpm at room temperature (final volume should be slightly less than 20µL)
4. Adjust volume to 21 µL
5. Store at -80°C if not immediately proceeding to the next step.

**First strand cDNA synthesis**

1. Assemble the following reaction in a 200uL thin wall PCR tube:

Random Hexamer Primers (3ug/µL, Invitrogen, #48190-011) 2 µL

mRNA 21µL

1. Incubate the sample in a PCR thermocycler at 65°C for 5 minutes, and then place the tube on ice.
2. Mix the following in order, make 10% extra reagent for multiple samples:

- 5X first strand buffer (Invitrogen, #18064-014) 8µL
- 100mM DTT (Invitrogen, #18064-014) 4µL
- dNTP mix (10mM) 2µL
- RNaseOUT (40U/µL) (Invitrogen, #10777-019) 1µL

1. Add 15µL of mixture to the tube, mix well, and heat the sample at 25°C in a thermocycler for 2 minutes.
2. Add 2µL SuperScript II (200U/ µL, Invitrogen, #18064-014) to the sample, and incubate the sample in a thermocycler with following program:

Step 1 25°C 10 min

Step 2 42°C 50 min

Step 3 70°C 15 min

Step 4 4 °C Hold

**Second strand cDNA synthesis**

- - 1. Put the tubes from section 2.3, step 5 on ice.
    2. Add 31µL of H_2_O to the first strand cDNA synthesis mix.
    3. Add the following reagents:
- 5X second strand buffer (Invitrogen #10812-014) 20µL
- dNTP mix (10mM) 3µL
  - 1. Mix well, incubate on ice 5 minutes or until well chilled, and add:
- RNaseH (2U/µL, Invitrogen, #18021-014) 1µL

##### DNA Pol I (10U/µL, Invitrogen, #18010-025) 5µL

- - 1. Mix well, and incubate at 16°C in a thermocycler for 2.5 hours.
    2. Purify the DNA with a QIAquick PCR spin column (Qiagen, #28106), and elute in 32µL of EB solution.

**End repair**

1. Prepare the following reaction mix:

- Eluted DNA 30µL
- H_2_O 45µL
- T4 DNA ligase buffer with 10mM ATP 10µL
- dNTP mix (10mM) 4µL
- T4 DNA polymerase (3U/µL) 5µL
- Klenow DNA polymerase (5U/µL) 1µL
- T4 PNK (10U/µL) 5µL

1. Incubate the sample at 20°C for 30 minutes.
2. Purify the DNA with a QIAquick PCR spin column (QIAGEN, #28106), and elute in 32µL of EB solution.

**Addition of a single <A> base**

1. Prepare the following reaction mix:

- Eluted DNA 32µL
- 10X NEBuffer 2 5µL
- dATP(1mM) 10µL
- Klenow 3’ to 5’ exo- (5U/µL) 3µL

1. Incubate the sample at 37°C in for 30min.
2. Purify the DNA with a QIAquick PCR Spin column and elute in 23µL of EB solution.

**Adaptor ligation (using Enzymatics ultrapure T4 DNA ligase)**

Prepare the following reaction mix:

- - Eluted DNA 23µL
  - 2X Rapid Ligation Buffer 25µL
- Adaptor oligo mix 1µL
- T4 DNA Ligase (600U/µL) 1µL

Incubate the sample at RT for 15min.

Purify the DNA with a QIAquick MinElute column (Qiagen, #28006), and elute in 10µL of EB solution. Any alcohol remaining in the eluate at this point may cause upwelling while loading the gel in the next step and lead to loss of sample. To ensure that all traces of alcohol in Buffer PE are removed from final eluate, place tubes at 37°C for 3-4 mins after spinning to remove residual Buffer PE, let cool to RT for 2-3 mins and add 13 µL Buffer EB.

**Gel purification of cDNA Templates**

Place BioRad ReadyAgarose gels in gel rig and fill with running buffer (1X TAE)

Use separate gels for each subject

Add 3 µL of 5X Loading Buffer to the sample

Load the sample into one well, and use both 50bp and 100bp DNA ladders as markers.

Run gel at 120V for 60min.

Stain for 30 mins in 1X SYBR Gold dye solution

Using blue light transillumination, cut a gel slice at 300 bp +25bp and purify the DNA using the QIAquick gel extraction kit (Qiagen, #28706).

Elute the DNA into 30µL of EB solution.

**PCR Enrichment of Purified cDNA Templates**

Set up PCR master mix, make 10% extra reagent for multiple samples, and aliquot 20µL to each PCR tube:

- 5X cloned Phusion Buffer (NEB, #F-530) 10µL
- PCR primer 1.1 1µL
- PCR primer 2.1 1µL
- 25mM dNTP mix 0.5µL
- Phusion polymerase (NEB, #F-530) 0.5µL
- H_2_O 7µL

Add 30µL of purified ligation mix from section 2.8, step 4 to the PCR tube.

Run following PCR cycle:

- 98°C 30 sec
- 98°C 10 sec
- 65°C 30 sec 15X
- 72°C 30 sec
- 72°C 5 min
- 4°C hold

Purify the DNA with a QIAquick column (Qiagen, #28106), and elute in 30µL of EB solution (I added 3 µL of 3M sodium acetate to the PCR rxn + Buffer PBI mix as the color changed to slightly less yellow after adding Buffer PBI).

Run 1 uL of the purified product on an Agilent Bioanalyzer DNA High-Sensitivity chip to QC the final product and quantify the DNA concentration by spectrophotometry.

**Appendix B**

**TopHat, Cufflinks and BEDTools settings used for bioinformatics processing**

1. Align reads using TopHat (version 1.0.14)

tophat -o $1.SE.TopHat.out -m 2 -p 8 ~/bin/bowtie/bowtie-0.12.1/indexes/hg18_inclusive <input.fastq>

1. Convert TopHat SAM file to BED file with each read identified by its start position

awk '{print $3,$4-1,$4,$1' <input.SAM> | sed 's/\s/\t/g' > output.bed

1. Download transcripts in the RefSeq database in BED12 file format from the Table browser at [www.genome.ucsc.edu](http://www.genome.ucsc.edu)
2. Convert the above BED12 file to BED6 format (one exon per line)

bed12ToBed6 -i <RefSeq BED12 file> > <output BED6 file>

1. Count reads mapping to individual exons

coverageBed -a <BED file from step 2> -b <BED6 file from step 3> > <output read count file>

All Perl scripts and BED files used for the above analyses are available from the authors upon request.

cuffdiff settings used for validation of alternative splicing results

cuffdiff -p 16 --FDR 0.5 --min-alignment-count 5 -M ./ribosomal_RNA.filter.gtf ./igenome.anemic.gtf 198564.LCL.PE51.tophat.bam,165583.LCL.PE51.tophat.bam,163383.LCL.PE51.tophat.bam,121424.LCL.PE51.tophat.bam,108971.LCL.PE51.tophat.bam,189793.LCL.PE51.tophat.bam,132306.LCL.PE51.tophat.bam,158592.LCL.PE51.tophat.bam 119657.LCL.PE51.tophat.bam,133237.LCL.PE51.tophat.bam,118655.LCL.PE51.tophat.bam,112916.LCL.PE51.tophat.bam,116099.LCL.PE51.tophat.bam,113235.LCL.PE51.tophat.bam,119522.LCL.PE51.tophat.bamAdditional Figure 1


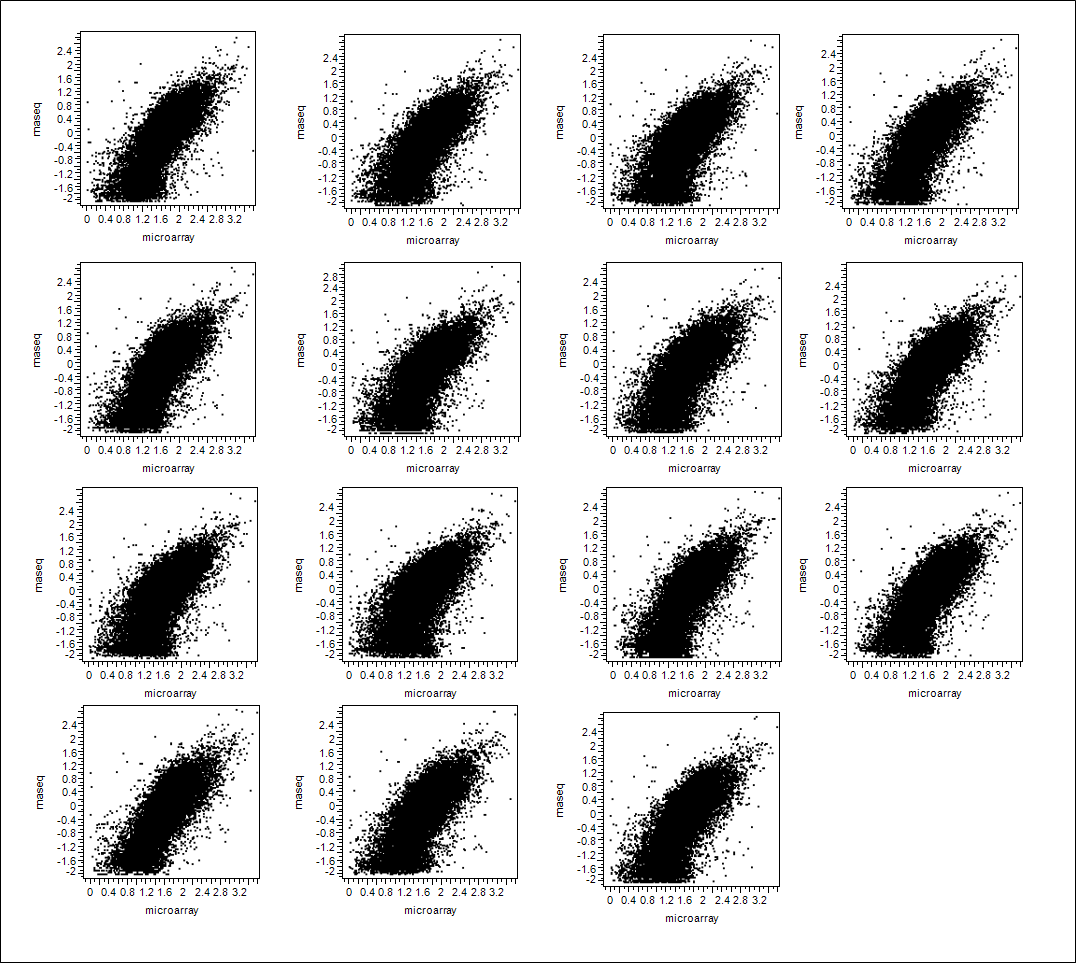


Correlation of RNA-Seq and microarray results for 16 lymphoblastoid cell lines

Additional Figure 2

**
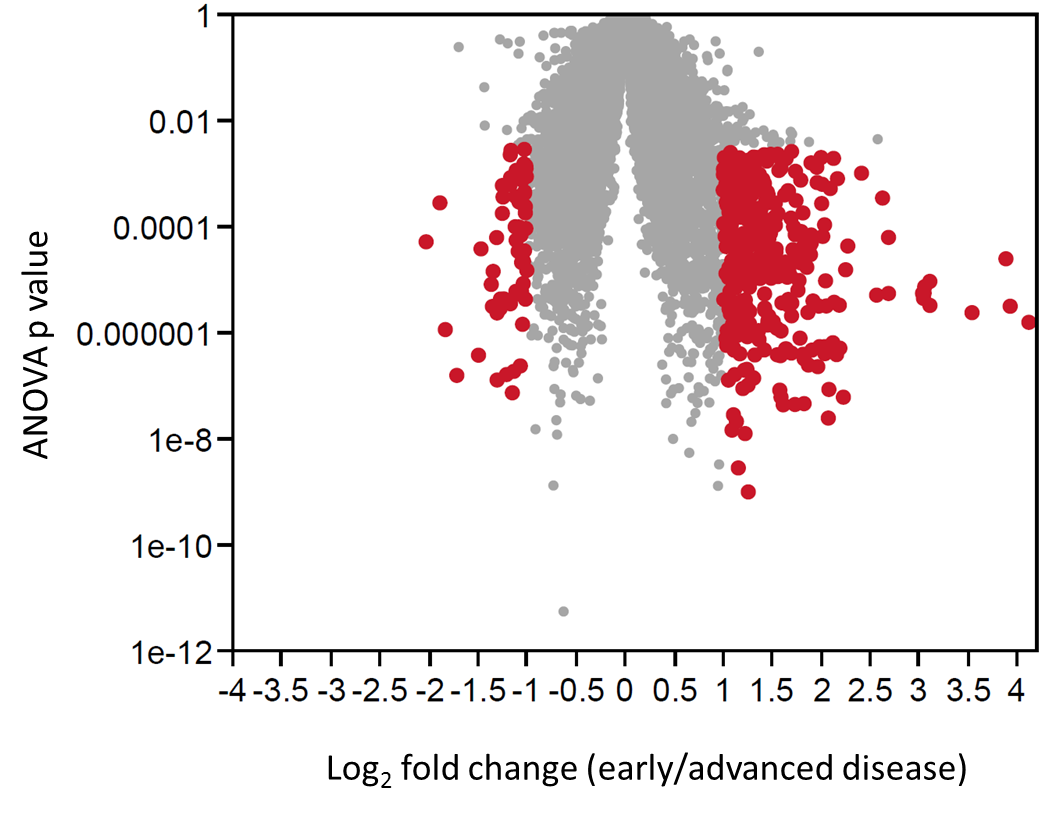
**

Volcano plot from Affymetrix U133 Plus 2 arrays on carotid artery RNA samples showing 4039 genes with fold change greater than 1.25 and p<0.05 after 10% FDR correction

Additional Figure 3

**
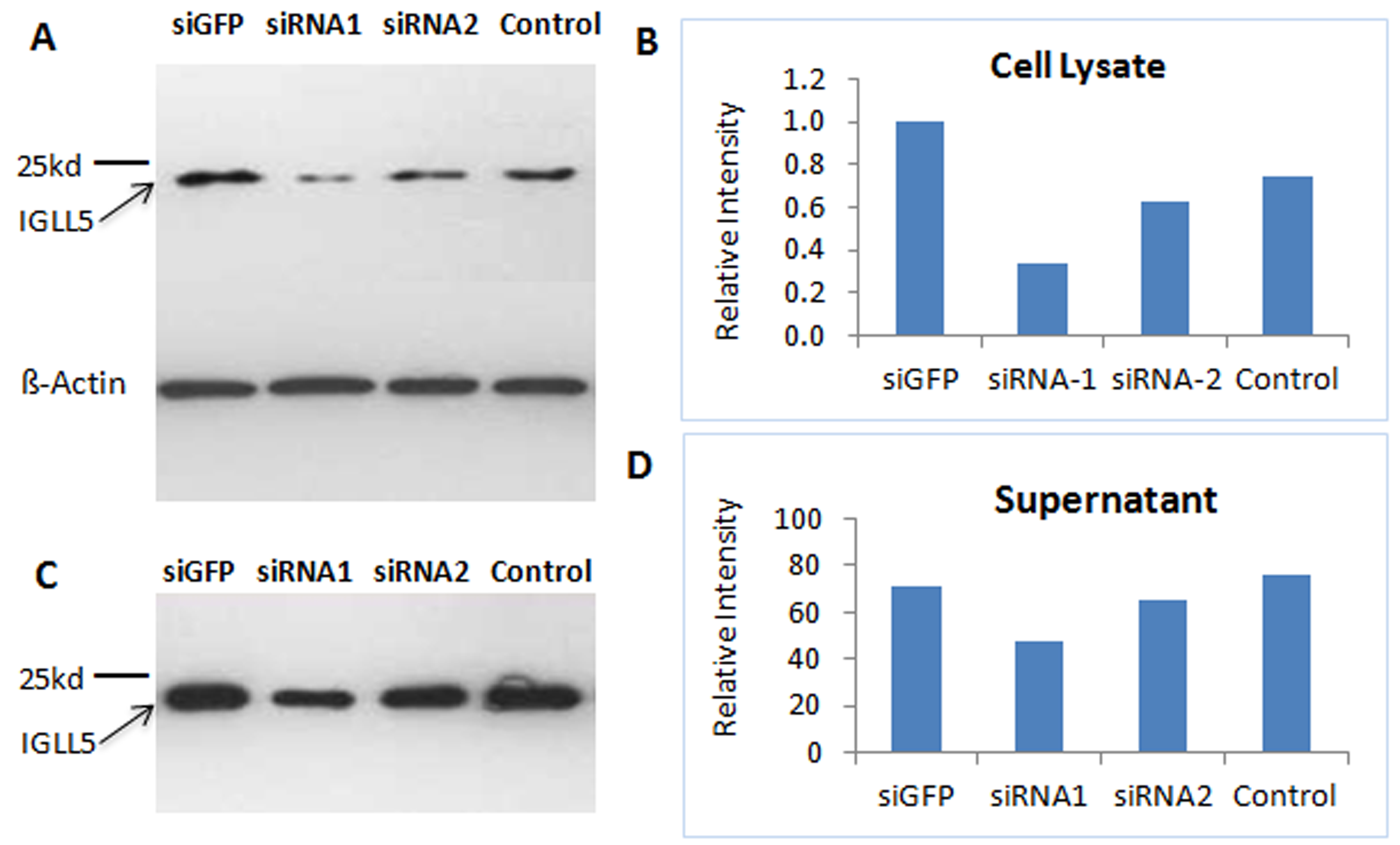
**

Western Blot of Immunoglobulin Lambda-Like Polypeptide 5 (IGLL5) protein after siRNA knockdown in human monocyte-derived macrophages. **A**: Cell lysate: siGLP (GFP-specific siRNA control), siRNA 1 (SI05459657), siRNA2 (SI05459664), and non-transfected control cells with respective β-actin control band. **B**: Relative band intensity of cell lysates. **C**: Corresponding Western Blot of cell supernatants. **D**: Relative band intensity of corresponding supernatants.

Additional Figure 4


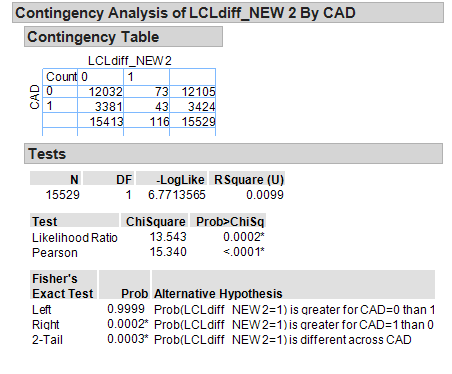


Fisher’s Exact Test results for CAD association enrichment in dysregulated genes

**Additional references**

1. Agatston AS, Janowitz WR, Hildner FJ, Zusmer NR, Viamonte M, Jr., Detrano R: **Quantification of coronary artery calcium using ultrafast computed tomography**. *J Am Coll Cardiol* 1990, **15**(4):827-832.

2. Bolton BJ, Spurr NK: **B-Lymphocytes**. In: *Culture of Immortalized Cells.* Edited by Freshney RI, Freshney MJ: John Wiley & Sons; 1996: 283-297.

3. Marioni JC, Mason CE, Mane SM, Stephens M, Gilad Y: **RNA-seq: an assessment of technical reproducibility and comparison with gene expression arrays**. *Genome Res* 2008, **18**(9):1509-1517.

4. Trapnell C, Pachter L, Salzberg SL: **TopHat: discovering splice junctions with RNA-Seq**. *Bioinformatics* 2009, **25**(9):1105-1111.

5. Quinlan AR, Hall IM: **BEDTools: a flexible suite of utilities for comparing genomic features**. *Bioinformatics* 2010, **26**(6):841-842.

6. Raghavachari N, Xu X, Harris A, Villagra J, Logun C, Barb J, Solomon MA, Suffredini AF, Danner RL, Kato G *et al*: **Amplified expression profiling of platelet transcriptome reveals changes in arginine metabolic pathways in patients with sickle cell disease**. *Circulation* 2007, **115**(12):1551-1562.
