# Supplementary material for: Identification of candidate genes involved in coronary artery calcification by transcriptome sequencing of cell lines
Source: BMC Genomics. 2014 Mar 14;15:198. doi: 10.1186/1471-2164-15-198 (PMC4003819; doi:10.1186/1471-2164-15-198)
Supplement: Additional file 3 — Ingenuity Pathways Analysis results Description: Complete output from Ingenuity Pathways Analysis. [file 1471-2164-15-198-S3.pdf]

IPA® Summary of Analysis

Analysis Name: LCL_revised_for_Abdel - 2012-08-01 09:04 AM
Analysis Creation Date: 2012-08-01
Build version: 162830
Content version: 12710793 (Release Date: 2012-05-07)

Analysis settings

View

Reference set: Ingenuity Knowledge Base (Genes Only)

Relationship to include: Direct and Indirect

Includes Endogenous Chemicals

Optional Analyses: My Pathways My List

Filter Summary:

Consider only relationships where

confidence = Experimentally Observed

Top Networks

ID	Associated Network Functions	Score
1	Cardiovascular System Development and Function, Organismal Development, Tissue Morphology	48
2	Lipid Metabolism, Small Molecule Biochemistry, Drug Metabolism	36
3	Cell Death, Cell Signaling, Nucleic Acid Metabolism	16

(c) 2000-2012 Ingenuity Systems, Inc. All rights reserved.

Top Bio Functions

Diseases and Disorders

Name	p-value	# Molecules
Cancer	1.18E-03 - 4.23E-02	5
Developmental Disorder	2.70E-03 - 3.45E-02	4
Hereditary Disorder	2.70E-03 - 3.45E-02	9
Neurological Disease	2.70E-03 - 4.49E-02	7
Auditory Disease	5.39E-03 - 5.39E-03	1

Molecular and Cellular Functions

Name	p-value	# Molecules
Cell-To-Cell Signaling and Interaction	6.17E-04 - 4.86E-02	9
Cell Morphology	1.09E-03 - 4.75E-02	14
Cell Cycle	2.70E-03 - 4.09E-02	6
Cell Death	2.70E-03 - 3.71E-02	4
Cellular Assembly and Organization	2.70E-03 - 4.03E-02	9

(c) 2000-2012 Ingenuity Systems, Inc. All rights reserved.

Physiological System Development and Function

Name	p-value	# Molecules
Cardiovascular System Development and Function	7.10E-06 - 4.49E-02	4
Organismal Development	7.10E-06 - 4.75E-02	9
Tissue Morphology	7.10E-06 - 4.49E-02	11
Connective Tissue Development and Function	2.70E-03 - 4.49E-02	3
Digestive System Development and Function	2.70E-03 - 4.75E-02	2

(c) 2000-2012 Ingenuity Systems, Inc. All rights reserved.

INGENUITY® SYSTEMS

Top Canonical Pathways

Name	p-value	Ratio
Tight Junction Signaling	8.35E-03	3/163 (0.018)
Calcium Signaling	1.2E-02	3/210 (0.014)
ERK5 Signaling	1.21E-02	2/64 (0.031)
Cholecystokinin/Gastrin-mediated Signaling	3.03E-02	2/106 (0.019)
Glycerolipid Metabolism	3.2E-02	2/156 (0.013)

Top Molecules

Fold Change up-regulated

Molecules	Exp. Value	Exp. Chart
IGLL1/IGLL5*	↑5.991	
DPYSL4	↑3.926	
DNAJC12*	↑2.563	
CKB	↑2.530	
TNFSF8	↑2.429	
TNC (includes EG:116640)	↑2.338	
TJP1 (includes EG:21872)*	↑2.192	
DGKG*	↑2.178	
GSTA4	↑2.177	
GAP43*	↑2.132	

(c) 2000-2012 Ingenuity Systems, Inc. All rights reserved.

INGENUITY® SYSTEMS

Fold Change down-regulated

Molecules	Exp. Value	Exp. Chart

Top Upstream Regulators

Upstream Regulator	p-value of overlap	Predicted Activation State
ZFYVE9	3.18E-05	
TGFB3	3.22E-05	
quinapril	7.92E-05	
HNRNPAB	2.36E-04	
EGF (includes EG:13645)	2.82E-04	

(c) 2000-2012 Ingenuity Systems, Inc. All rights reserved.

Top My Lists

Name	p-value	Ratio

Top My Pathways

Name	p-value	Ratio
YS 022210	1E-01	1/48 (0.021)

Top Tox Lists

Name	p-value	Ratio
Acute Renal Failure Panel (Rat)	1.21E-02	2/62 (0.032)
Cytochrome P450 Panel - Substrate is an Eicosanoid (Rat)	1.34E-02	1/5 (0.2)
Cytochrome P450 Panel - Substrate is an Eicosanoid (Mouse)	1.61E-02	1/6 (0.167)
Cytochrome P450 Panel - Substrate is an Eicosanoid (Human)	1.87E-02	1/7 (0.143)
Hepatic Fibrosis	2.2E-02	2/85 (0.024)

(c) 2000-2012 Ingenuity Systems, Inc. All rights reserved.

Top Tox Functions

Assays: Clinical Chemistry and Hematology

Name	p-value	# Molecules
Increased Levels of Creatinine	3.71E-02 - 3.71E-02	1

Cardiotoxicity

Name	p-value	# Molecules
Cardiac Congestive Cardiac Failure	2.27E-01 - 2.27E-01	1
Heart Failure	2.27E-01 - 2.27E-01	1
Cardiac Hypertrophy	2.46E-01 - 2.78E-01	1
Cardiac Necrosis/Cell Death	3.42E-01 - 3.42E-01	1
Cardiac Arteriopathy	4.90E-01 - 4.90E-01	1

Hepatotoxicity

Name	p-value	# Molecules
Glutathione Depletion In Liver	5.01E-02 - 5.01E-02	1
Liver Hemorrhaging	6.79E-02 - 6.79E-02	1

(c) 2000-2012 Ingenuity Systems, Inc. All rights reserved.

INGENUITY® SYSTEMS

Liver Damage	2.33E-01 - 2.33E-01	1
Liver Necrosis/Cell Death	3.29E-01 - 3.29E-01	1

Nephrotoxicity

Name	p-value	# Molecules
Renal Necrosis/Cell Death	2.70E-03 - 4.34E-01	3
Glomerular Injury	5.39E-03 - 5.39E-03	1
Renal Dilation	5.39E-03 - 5.39E-03	1
Kidney Failure	1.87E-02 - 3.14E-02	2
Renal Fibrosis	1.87E-02 - 1.87E-02	1

(c) 2000-2012 Ingenuity Systems, Inc. All rights reserved.
